# Critical Points in the Management of Pseudohypoaldosteronism Type 1

**DOI:** 10.4274/jcrpe.v3i2.20

**Published:** 2011-06-08

**Authors:** Tülay Güran, Serpil Değirmenci, İpek K. Bulut, Aysun Say, Felix G. Riepe, Ömer Güran

**Affiliations:** 1 Zeynep Kamil Maternity and Childrens’ Diseases Training and Research Hospital, Division of Pediatric Endocrinology and Diabetes, Istanbul, Turkey; 2 Sisli Etfal Training and Research Hospital, Division of Neonatal Intensive Care Unit, Istanbul, Turkey; 3 Zeynep Kamil Maternity and Childrens’ Diseases Training and Research Hospital, Division of Pediatric Nephrology, Istanbul, Turkey; 4 Zeynep Kamil Maternity and Childrens’ Diseases Training and Research Hospital, Division of Neonatal Intensive Care Unit, Istanbul, Turkey; 5 Christian-Albrechts University, Division of Pediatric Endocrinology and Diabetes, Kiel, Germany

**Keywords:** Pseudohypoaldosteronism type 1, treatment, pitfalls

## Abstract

Pseudohypoaldosteronism type 1 (PHA-1, MIM #264350) is caused by defective transepithelial sodium transport. Affected patients develop life-threatening neonatal-onset salt loss, hyperkalemia, acidosis, and elevated aldosterone levels due to end-organ resistance to aldosterone. In this report, we present a patient diagnosed as PHA-1 who had clinical and laboratory findings compatible with the diagnosis and had genetically proven autosomal recessive PHA-1. The patient received high doses of sodium supplementation and potassium-lowering therapies; however, several difficulties were encountered in the management of this case. The aim of this presentation was to point out the potential pitfalls in the treatment of such patients in the clinical practice and to recommend solutions.

**Conflict of interest:**None declared.

## INTRODUCTION

Pseudohypoaldosteronism type 1 (PHA-1, MIM #264350) is a rare disease caused by defective transepithelial sodium transport. Affected patients develop life-threatening, neonatal-onset salt loss, hyperkalemia, acidosis, and elevated aldosterone levels due to end-organ resistance to aldosterone. Accordingly, they are insensitive to mineralocorticoid treatment, but respond to high doses of sodium supplementation and potassium-lowering therapies. 

A multidisciplinary team including a neonatologist, an endocrinologist, a neurologist and a dietician is essential for evaluation of longitudinal growth and neurological development in PHA-1 patients. We would like to share our experience on the difficulties of the management of a patient diagnosed in the newborn period with genetically proven autosomal recessive PHA-1. 

## CASE REPORTS

A seven-day-old female baby was brought to the emergency department because of jaundice.  The family did not report any symptoms other than jaundice. The baby was born to third-degree consanguineous parents at term.  Birth weight was 3060 g. The baby was reported to be apparently healthy during the first 6 days of life. 

Physical examination was unremarkable except for mild lethargy. External genitalia were normal. Initial biochemistry revealed an elevated serum indirect bilirubin (24 mg/dL), but unexpectedly abnormal serum electrolytes (Na: 113 mEq/L and K: 13 mEq/L), confirmed by double testing. Results of urinanalysis, renal functions and renal ultrasound were normal. Pelvic ultrasound showed normal Mullerian structures. 

The infant was admitted to the neonatal intensive care unit for hyperbilirubinemia and abnormal electrolytes discordant with her general well-being.  Intravenous (IV) fluid and sodium replacement as well as potassium-lowering therapy were started immediately. Within the following 2-3 hours, the patient deteriorated rapidly, developed cardiac arrest and needed  cardiopulmonary resuscitation. The baby  improved with the above-mentioned aggressive supportive therapy and was  discharged at 1.5 months of age to be followed on an outpatient basis. The patient was diagnosed as a case of autosomal recessive PHA-1. This diagnosis was established because of absent glucocorticoid deficiency, very high aldosterone and renin levels and elevated sweat, salivary and urinary sodium concentrations [serum aldosteron: 2782.3 pg/ml (normal: 10-160); renin: 170 ng/ml/hr (normal: 0.5-1.19); sweat sodium 75 mEq/L, saliva sodium: 153 mEq/L, urinary sodium: 94 mEq/L]. The diagnosis was confirmed by genetic analysis showing a novel IVS8-2A>G mutation in the SCNN1A gene.

## DISCUSSION

PHA-1 is a rare disease caused by defective transepithelial sodium transport due to mutations in the genes encoding a (SCNN1A), b (SCNN1B) or g (SCNN1G) subunit of the epithelial Na+ channel (ENaC). Affected patients develop life-threatening, neonatal-onset salt loss, hyperkalemia, acidosis, and elevated aldosterone levels due to end-organ resistance to aldosterone. Affected patients require life-long salt supplementation and dietary manipulations aimed at reducing potassium levels (1).

We would like to summarize our observations and the pitfalls we have encountered in the management of this patient. The initial phase of treatment during the first 1.5 months of life required almost continuous IV fluid administration for the correction of dehydration, severe electrolyte imbalances and metabolic acidosis. A fluid intake up to 350 mL/kg/day was needed, varying in accordance with the degree of dehydration. Hyponatremia was corrected by IV therapy (sodium concentration increased to provide up to 50 mEq/kg/day). IV sodium bicarbonate (NaHCO3) infusions up to 8 mEq/kg/day were administered to correct metabolic acidosis and to improve severe hyperkalemia. Besides sodium polystyrene sulphonate resin therapy (6 g/kg/day in 8 doses), sodium bicarbonate, calcium carbonate and glucose-insulin infusions (as needed), transient peritoneal dialysis (two occasions) for severe hyperkalemia became necessary. The general well-being of our patient was not disturbed although the serum potassium levels increased to 10-12 mEq/L. However, the patient developed cardiac arrest 3-4 hours after admission despite all the supportive therapy. Therefore, we concluded that the team should be ready to initiate all invasive and non-invasive measures, including dialysis preparations, as soon as the first signs of salt-wasting crisis begin. The transient peritoneal dialysis catheter may be maintained until the electrolyte values stabilize.

In the follow-up of this patient, we have observed flares of pustules especially in the dependent parts of the body just before and during the severe salt-wasting attacks. Indeed, patients with PHA-1 may present with seborrheic dermatitis, folliculitis or miliaria rubra-like lesions preceding severe salt-wasting crises. There is a hypothesis that the secretion of sweat with elevated sodium chloride concentrations directly affects the eccrine ducts (2). This finding should alert physicians and families about impending salt-wasting crises to take preventive measures.

After discharge at 1.5 months of age, the infant was maintained on oral intake only. The mainstay of the treatment in PHA-1 should include sodium chloride supplements plus cation-exchange resins. Our patient required very high maintenance doses of sodium chloride, occasionally increased up to 50 mEq/kg/day and divided in 6-8 doses, and cation-exchange resins (6 g/kg/day in 4-6 doses). In these patients, close serum electrolyte monitoring is needed in the follow-up. At least twice daily serum electrolyte control is fundamental during the salt-losing crises. Metabolic acidosis can be managed by oral NaHCO3. Our patient also required such treatment (2 mEq/kg/day NaHCO3 in 4 doses), which helps to improve the low sodium levels besides correcting the acidosis. Monitoring of blood pH is essential for the regulation of the NaHCO3 dosage. Because of the commercial unavailability of liquid oral sodium chloride preparations,we had to use table salt (1 gram of table salt contains approximately 17 mEq sodium) that was added as fractionated doses to the formula of the baby. Babies may not tolerate this salt-added formula, they show their dislike and even may cough or vomit. We have overcome this problem by feeding the baby upright and giving this food at a time when she appeared to be most hungry.

We had also problems with cation-exchange resins. Potassium-binding resins that are commercially available generally include sodium and calcium polystyrene sulphonate. Sodium containing ion-exchange resins should be preferred both to improve sodium deficit and to prevent iatrogenic hypercalcemia and hypercalciuria that can be seen in PHA-1 patients (3). Calcium-containing exchange resins may augment hypercalciuria and hypercalcemia in PHA-1 patients, especially when administered in high doses. As a second point of consideration, they are generally unpalatable and patients with PHA-1 generally need these drugs in high doses as a life-long maintenance treatment. In the newborn period, it is recommended that potassium polystyrene drugs be administered as rectal enema, since oral administration may cause bowel obstruction. On the other hand, particular care is needed with rectal administration as excessive dosage or inadequate dilution could result in impaction of the resin, intestinal haemorrhage or in colonic necrosis. This enema should be retained for at least nine hours. Afterwards the colon needs to be irrigated to remove the resin. These facts render both rectal and oral routes unsuitable for such babies. Once the mixture has been prepared, it should be used straight away. In our patient, we had to increase the dose of sodium polystyrene to as high as 16 g/day given in 8 doses in the newborn period. Rectal enema at this dosage caused rectal prolapse in our patient. To overcome the problems with usage of cation-exchange resins and to manage dangerous levels of plasma potassium, we had to alternate the administration of the resin enterally either by mouth or by nasogastric tube or as rectal enema.  We had to be extremely careful in oral or rectal administration to use proper dilutions according to the baby’s tolerance. We were usually able to administer the drug by mouth as a suspension in a small amount of water (3-4 mL per gram of powder), or in a sweetened liquid (but not fruit juices, which contain potassium). The baby was positioned carefully when ingesting the resin, in order to avoid aspiration, which might lead to bronchopulmonary complications. 

Feeding of babies with PHA-1 is also problematic. In weaning the baby from breastfeeding, the use of commercial formulas makes it very difficult to reduce the potassium content of the infant’s diet below 1-2 mEq/kg/day. Comprehensive evaluation by a professional dietician for a low-potassium diet both during the weaning period and later with supplementary foods is required. Growth failure is frequent in these patients.  

Outpatient follow-up of such patients should inevitably involve close contact with the family, especially during infancy, for monitoring of general health, electrolytes and weight gain.  This can be accomplished both by clinical visits and through phone calls or online contact.  Our patient is currently 6 months old. We have followed her monthly in the clinic and weekly via phone-call or internet. These intervals have been more frequent during periods of acute illness.

The monitoring of growth and neurological development by a multidisciplinary team is also an important aspect of the follow-up of PHA-1 patients. At six months of age, our patient showed a satisfactory physical and neuromotor development in accordance with respective age references. 
